# Hydroxytyrosol recovery from olive pomace: a simple process using olive mill industrial equipment and membrane technology

**DOI:** 10.1007/s13197-023-05832-x

**Published:** 2023-09-20

**Authors:** Maria F. C. Romeu, Jorge Bernardo, Carla I. Daniel, Nuno Costa, João G. Crespo, Luís Silva Pinto, Manuel Nunes da Ponte, Ana V. M. Nunes

**Affiliations:** 1 Zeyton Nutraceuticals, Parque Industrial do Penique, Estrada Nacional 2, Km 585, Odivelas, Ferreira do Alentejo, Portugal; 2 LAQV, REQUIMTE, Departamento de Química, Faculdade de Ciências e Tecnologia, Universidade Nova de Lisboa, 2829-516 Caparica, Portugal; 3 Azal Azeites do Alentejo, Estrada Nacional 254, 7170-107 Redondo, Portugal

**Keywords:** Olive pomace, Olive polyphenols, Hydroxytyrosol, Tyrosol, Nanofiltration, Reverse osmosis

## Abstract

In this work, pilot-scale nanofiltration was used to obtain aqueous solutions rich in hydroxytyrosol and tyrosol from olive oil by-products. A large-scale simple process involving olive mill standard machinery (blender and decanter) was used for the olive pomace pre-treatment with water. The aqueous extract was then directly fed to a nanofiltration unit and concentrated by reverse osmosis. Final concentration factors ranged between 7 and 9 for hydroxytyrosol and between 4 and 7 for tyrosol. The final aqueous solution, obtained as retentate stream of reverse osmosis, was highly concentrated in hydroxytyrosol and tyrosol and their concentrations remained stable over at least 14 months.

## Introduction

The Mediterranean diet has been linked to the prevention of cardiovascular diseases (Tong et al. 2016). As olive oil is its most common fat, its potential benefits in the health of consumers have attracted a lot of attention. Although olive polyphenols are a minor fraction of the compounds present in olive oil, they are thought to play a key role in many biological interactions (Serreli and Deiana 2020). Specifically, hydroxytyrosol, tyrosol and their derivatives have been object of several studies highlighting protective effects toward cardiovascular health (Covas et al. 2006a, b; Karković Marković et al. 2019). Covas et al. (2006a, b) reported a clinical study on olive oil consumption involving 200 volunteers, for which oxidized LDL levels decreased with increasing polyphenol concentration in a dose-responsive manner. Based mainly on this study, the European Food Safety Authority has approved the following health claim for olive oils with high content of hydroxytyrol, tyrosol and their derivatives: “Olive oil polyphenols contribute to the protection of blood lipids from oxidative stress” (EFSA Panel 2011). The growing interest in these compounds has prompted the development of methods for the separation, concentration and purification of these substances and their derivatives from various materials derived from the olive tree, especially those with less economic value, such as olive pomace and olive mill wastewaters. Indeed, only 2% of the phenolic fraction found in the olive fruit ends in olive oil, while 98% are retained in the sub-products (Obied et al. 2005).

Olive pomace or olive mill wastewaters are highly polluting effluents, and many different processes have been proposed for their treatment. Membrane-based processes have been pointed out as among the most suitable and sustainable ones for this purpose (Zagklis et al. 2013). Recent reviews summarise the work done on integrated processes, where membranes were used for olive mill effluent treatment, but also for the separation of olive polyphenols (Gebreyohannes et al. 2016; Bazzarelli et al. 2016; Castro-Muñoz and Fíla 2018; Cassano et al. 2021; Tapia-Quirós et al. 2022a).

These studies normally use a cascade of membrane processes, starting with microfiltration or ultrafiltration, and proceeding with nanofiltration, followed by reverse osmosis, obtaining thus several fractions with different molecules. As raw material, most of them use waste waters originated by traditional mills, which follow the so-called three-phase process to obtain olive oil from olives. Due to the large amount of contaminated waters produced by this process, it has been progressively replaced, in modern mills, by a two-phase process, which produces one sole effluent (apart from the oil). This olive mill solid waste is a wet semi-solid paste, often referred to as pomace or *alperujo* (in Spanish). Using membranes to separate polyphenols from this type of effluent requires a pre-treatment.

Sygouni et al. (2019) have recently addressed this problem and developed a process whereby they submit the olive mill solid residue to extraction with water. In their pilot plant experiments, they use a 2.5 water to solid ratio. Subsequently, they apply ultrafiltration, nanofiltration and reverse osmosis to the aqueous solution, following very closely the process developed in the same laboratory by Zagklis and Paraskeva (2014) for olive mill wastewaters. In particular, they used nanofiltration membranes with a molecular cut-off of 800 Dalton and reverse osmosis with a 99% rejection of monovalent salts. The total phenolic content of the feed solution is distributed among the retentates of the three membrane processes, with only a very minor amount leaving the process in the reverse osmosis permeate. Although the authors present only values for total phenolic content of each fraction, they assume that the reverse osmosis retentate, the last in their series, is mainly composed of the smaller alcohols in their feed, that is, mainly hydroxytyrosol and tyrosol. The phenolic concentration in this fraction was 225 mg/L, corresponding to 8% of the total phenolic content of the feed.

Based on this work, Zagklis et al. (2015) and Zagklis and Paraskeva (2020) have recently designed a membrane multipurpose plant for the separation of phenolics from several sources, complemented with an adsorption–desorption process. The plant design uses olive mill wastewaters as the raw-materials, but it could be adapted to the two-phase olive mill solid waste referred to above. Furthermore, the authors performed an extensive techno-economic study and concluded that the high value and market demand for natural ingredients justifies the investment and operational costs of this kind of separation process, making it economically viable (Zagklis et al. 2021).

Bellumori et al. (2018) used Italian olive mill wastewaters produced in 3 year campaigns (2013–2015) and ran them through a succession of membrane filtration processes—microfiltration, ultrafiltration, nanofiltration and reverse osmosis, followed by a concentration step in a vacuum evaporator of the retentates in each filtration. They thus obtained a variety of process streams, and analysed them for hydroxytyrosol, tyrosol, verbascoside and total phenols.

Tapia-Quirós et al. (2022b, 2022c) have proposed an integrated scheme for olive pomace processing, consisting of a preliminary extraction with water followed by sequential membrane-based fractionation steps and a final concentration step using reverse osmosis. The authors highlight that NF membranes allowed to separate between families of polyphenols due to their low rejection for low molecular weight polyphenols (Tapia-Quirós et al. 2022d).

When using olive mill effluents to recover phenolics, care should be taken to characterize the composition of these effluents, as it varies enormously over time. The main phenolic components of olives are glycosylated—oleuropein and ligstroside, but they suffer a series of enzymatic and chemical transformations during the maturation of the fruit, which continues in the effluents after olive oil production, as described by Obied et al. (2005, 2008). In particular, during storage, olive pomace and olive mill wastewater are enriched in hydroxytyrosol and tyrosol free molecules (Feki et al. 2006), which are the final products of those transformations.

The focus of this work was to produce concentrated solutions of hydroxytyrosol and tyrosol from olive mill solid waste (olive pomace) in an industrial setting, using nanofiltration, and reverse osmosis for concentration. A simpler process scheme than most described in the literature was explored in this work, as nanofiltration was the only separation step. Experiments were performed in the premises of an actual olive mill, using their standard machinery to pre-treat the olive pomace, right after the campaigns of 2014, starting from January 2015, and of 2015, starting January 2016 (each year, the olive harvesting and olive oil production campaign typically lasts from October to January). The process includes a pre-treatment, using the same equipment as is used in the two-phase olive mill to obtain olive oil. Olive pomace stored during the olive oil campaign was mixed with water in an industrial blender, and the mixture was run through a decanter, which is the same as used during to production of the oil. The resulting aqueous solutions were then fractionated directly in a nanofiltration unit, and the permeate concentrated by reverse osmosis. Pilot-scale nanofiltration and reverse osmosis units were installed in the olive oil mill for that purpose. Olive pomaces from different season crops were also characterized and the final hydroxytyrosol aqueous solutions (the retentates of reverse osmosis) analysed and investigated for stability over 14 months.

The novelty of the work lies mainly in the use of nanofiltration directly on the aqueous extract of the olive pomace. Contrarily, the other processes described in the literature use previously several membrane filtration steps, normally micro and ultrafiltration, with loss of hydroxytyrosol and tyrosol in the retentates of those steps. Moreover, the process described here was performed in an industrially relevant environment, an olive mill, providing a technology validation. Part of the process was actually performed using some of the equipment of the mill, namely the mixing blender and the two-phase decanter, which is reported in this work for the first time. This kind of product has application in several rapidly growing industry sectors, like food, cosmetics, pharmaceuticals, and nutraceuticals production industries.

## Materials and methods

### Chemicals

Hydroxytyrosol (≥ 98% mol purity) was purchased from Extrasynthese (Genay, France) and tyrosol (98% mol purity) was purchased from Aldrich (Steinheim, Germany) and were used as standards in liquid chromatography.

### Production of aqueous extracts from olive pomace

**Fig. 1:**
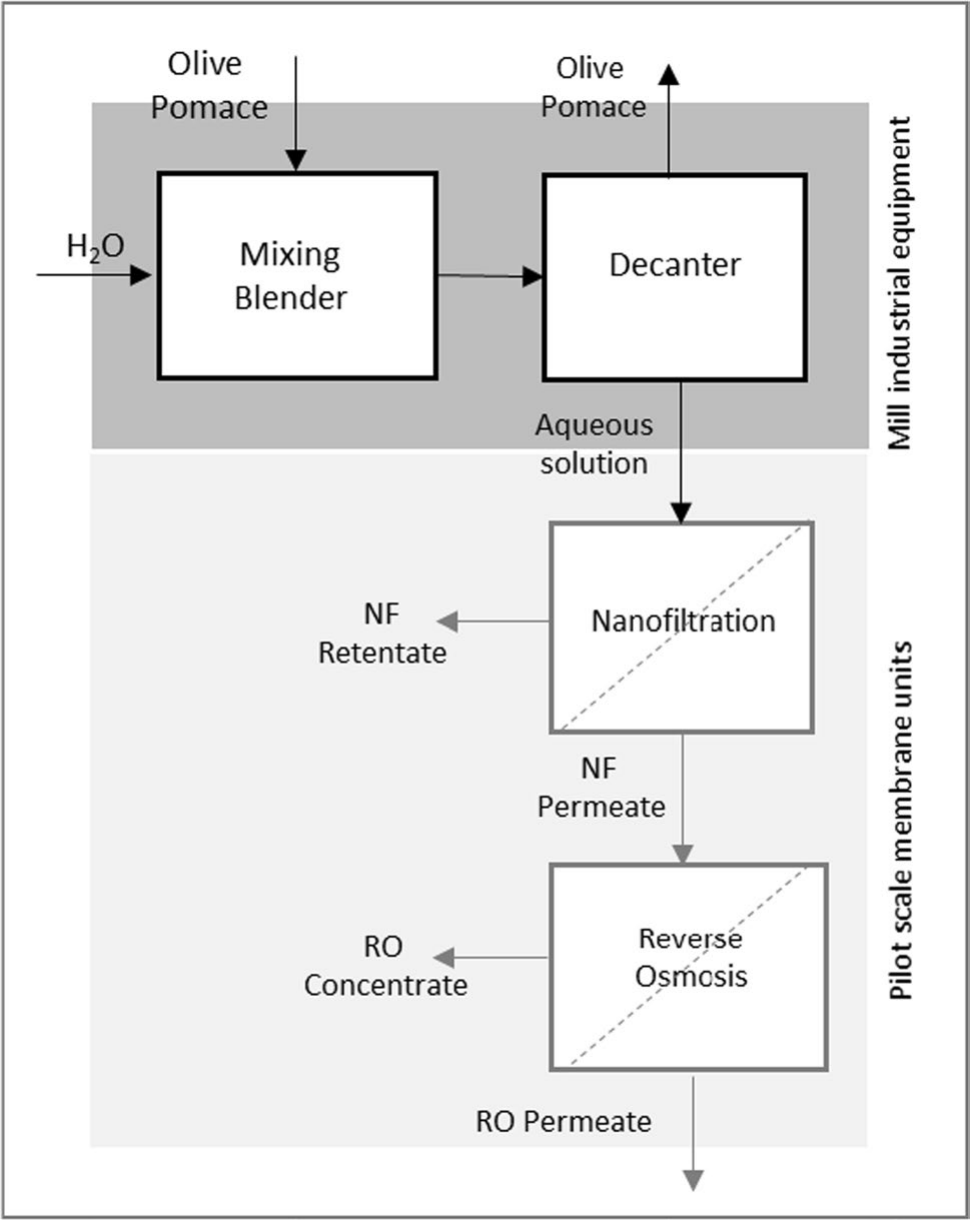
Schematic illustration of the process used during this work for olive pomace processing. Dark grey: olive mill industrial standard equipment; light grey: pilot scale membrane units. The product of the process is the RO concentrate

Aqueous extracts of olive pomace were produced at the actual premises of a commercial olive mill (AZAL Azeites do Alentejo, Redondo, Portugal), as illustrated in Fig. 1.

This extraction pre-treatment aimed at obtaining concentrated aqueous solutions of hydroxytyrosol and tyrosol (Katsinas et al. 2021). We used a low water to pomace mass ratio (2/3) for this purpose, in order to optimise this extraction step in relation to concentration of the extract and not to total quantity extracted from the pomace. This procedure is an economical viable option when there is unlimited availability of raw material.

A batch process was used, with successive operations carried out in the mill equipment where olives are usually processed to yield olive oil. These were a mixing blender, where usually the olive paste obtained by grinding is mixed, and a decanter, where the thoroughly homogenised olive paste is subject to centrifugation and the oil is separated from a wet solid.

The process started by contacting 1500 kg of olive pomace with 1000 L of water in the blender (Palacín H4,5). The aqueous phase was then separated from the semi-solid pomace phase using the mill decanter (Flottweg). It was then stored in a tank for subsequent processing by membrane technology.

The nanofiltration equipment is a pilot scale unit, equipped with a spiral-wound NF270-Filmtec membrane, with an active area of 7.5 m^2^. It includes feed and permeate tanks of 1000 L capacity each, a high-pressure diaphragm pump, a permeate flowmeter, and a 70 µm metallic pre-filter. Experiments were conducted using a concentration batch mode, where the retentate is recovered to the feed tank and the permeate collected individually (Sanches et al. 2017). The transmembrane pressure used was 10 bar, at the room temperature.

The nanofiltration permeate is subsequently concentrated by reverse osmosis, in a unit equipped with two spiral wound membrane modules (SW3040-40 Lenntech—Dow), with a total active area of 7 m^2^, at transmembrane pressures of approximately 50 bar. The retentate of the reverse osmosis process is the final product. It is an aqueous solution containing phenolic compounds, mainly hydroxytyrosol and tyrosol, carbohydrates, organic acids and salts.

### Analyses

The quantification of hydroxytyrosol and tyrosol in the various aqueous solutions obtained along the process were performed by HPLC, with a Dionex ICS3000 with the software Chromeleon. The column used was a non polar Waters Novapak C18 (150 × 3.9 mm) and the temperature was kept at 25 °C over the analysis time. The polar mobile phase was a mixture of 10% of methanol and 2% of acetic acid in water. The detection and quantification were made with a UV detector at 210 nm and 280 nm, using standard solutions of hydroxytyrosol and tyrosol (Miralles et al. 2014).

For the analysis of olive pomace content over time, 20 g of olive pomace were weighed, mixed with 13 mL of water and stirred at 300–400 rpm at room temperature over 1 h. After this initial treatment, the samples were centrifuged at 2000–3000 rpm over 15 min at room temperature. The aqueous phase was collected and analysed by HPLC. It is important to notice that this laboratory method tries to mimic the industrial process used to provide the feed of the nanofiltration, where three parts of pomace are contacted with two parts of water, as described above in “Production of aqueous extracts from olive pomace” section. The results of these analyses may not correspond to the total concentration in the pomace, but only to the expected concentrations of feed in the process used here.

## Results and discussion

### Hydroxytyrosol content of different olive pomaces during storage

In this work, olive pomace from the so-called two-phase processing of olives was used to produce a solution rich in olive polyphenols, namely hydroxytyrosol and tyrosol. The process consists in performing an aqueous extraction from the pomace, followed by a nanofiltration step and a final concentration using reverse osmosis. The nanofiltration membrane (NF270) used has a molecular weight cut-off of approximately 300 Da (Lopes-Muñoz et al. 2009). This retains higher molecular weight compounds, but exhibits high permeability towards hydroxytyrosol and tyrosol free molecules (molar mass 154 and 138 g mol^−1^, respectively), the main targets of this work.

As these alcohols appear in olives mostly connected through ester bonds to other molecules, and not in their free form, it is important to know the kinetics of enzymatic and acid transformation, through hydrolysis, into free molecules. The concentration of hydroxytyrosol and tyrosol in the nanofiltration permeate depends directly on their concentration as free molecules in the feed. An important parameter is therefore the phenolic composition of the olive pomace extract, as it varies not only with season crop and the type of olive tree variety (Pereira et al. 2020), but also with time elapsed since the olive oil production time (Feki et al. 2006).

**Fig. 2:**
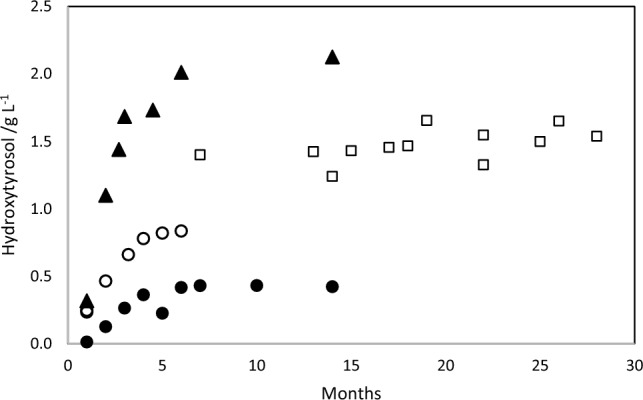
Free hydroxytyrosol concentration in aqueous solutions extracted from olive pomaces from three olive harvest seasons—2013 open square; 2014 open circle, filled circle; 2015 filed triangle

The increase in free hydroxytyrosol concentration of olive pomace from the harvest seasons of 2013, 2014 and 2015 was monitored over time. Olive pomaces from two-phase olive oil extraction system were collected and analysed for their content in free hydroxytyrosol. The olive pomace samples were processed as described in “Analyses” section and the resulting aqueous solutions analysed by HPLC. Results are presented in Fig. 2.

The concentration is plotted as function of time, measured in months since the end of December for each year (for 2013, pomaces were collected only in July 2014). The two different sets of values for 2014 refer to two different origins of the pomaces.

These results show essentially two features: (1) the concentration of free hydroxytyrosol in the aqueous solutions extracted from olive pomaces starts at virtually zero at the time of olive oil production, increases gradually during about 5 months, and reach a limiting value that remains stable for a long time; (2) this concentration limit varies from year to year, and from origin within the same year.

These findings are in good agreement with the work of Feki et al. (2006), who studied the effect of storage of olive mill wastewaters on hydroxytyrosol concentration. They found a noticeable increase in the hydroxytyrosol concentration after 5 months of storage, but noticed that the concentration of some of the other phenolic compounds in the wastewaters were significantly decreased. They attributed these simultaneous changes to the hydrolysis of oligomeric polyphenols that are hydroxytyrosol derivatives. Although their concentration values cannot be directly compared with ours, because they refer to wastewaters obtained by the traditional three phase olive mills, their findings are consistent with our results plotted in Fig. 2, where free hydroxytyrosol grows from close to zero for freshly obtained pomace to a limiting value at approximately 5 months afterwards. This limiting value probably represents the majority of the initial concentration of hydroxytyrosol derivatives existing in the waters extracted from fresh pomace.

Several publications have examined the variability of polyphenol content with origin and harvest year. For instance, Vinha et al. (2005) observed large differences in polyphenol content in Portuguese olives of different cultivar and geographical origin, and Pereira et al. (2020) examined monovarietal olive oils of the harvest of 2018, finding also large differences in hydroxytyrosol and tyrosol concentration. Results presented in Fig. 2 also show significant differences between seasons in the concentrations of HT and TY, indicating the existence of considerable variability.

### Nanofiltration and reverse osmosis experiments

The filtration of the aqueous solutions obtained by water extraction from olive pomace, as described in “Production of aqueous extracts from olive pomace” section, started with preliminary optimisation experiments. 1500 kg of pomace were mixed with 1000 kg of water in the olive mill blender, varying temperature (24–30 °C) and time of mixing (1–6 h). The conditions during the subsequent operation in the decanter were also varied, so that the volumes of the resulting water solution would vary from 1000 to 1600 L. The objective was to determine which water solutions would produce less fouling in the nanofiltration membrane, allowing higher throughput. From those experiments, it was concluded that lower time of mixing and lower temperature in the mixer and higher volume of water centrifuged from the pomace solid material in the decanter were the preferable conditions.

**Fig. 3:**
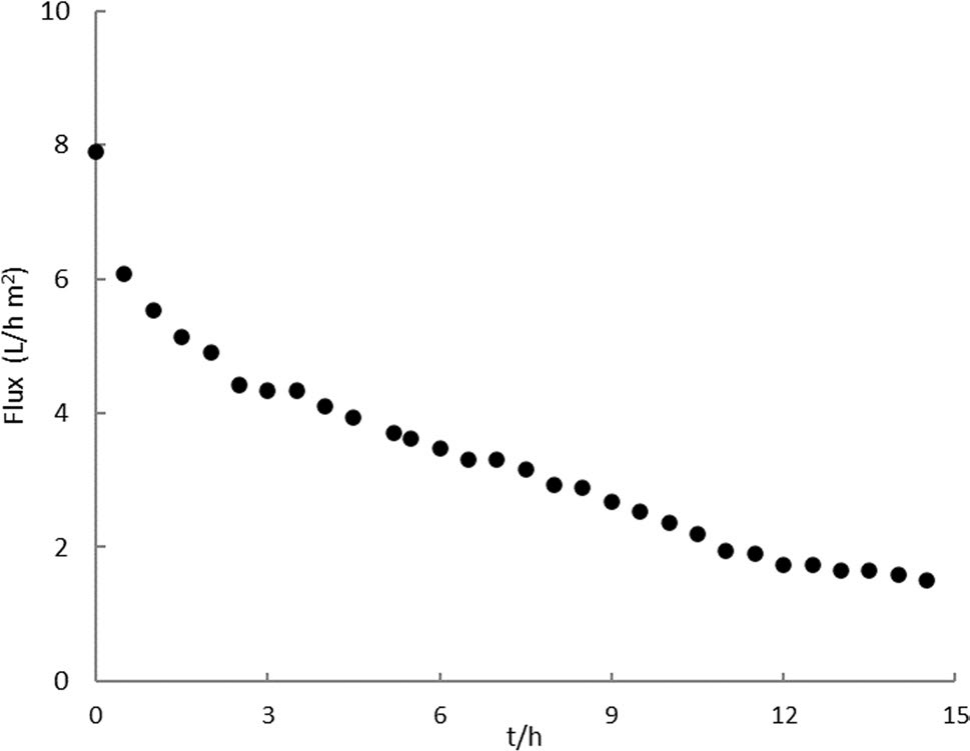
Permeate flux as a function of time

In Fig. 3, the permeability fluxes of a nanofiltration experiment are plotted as a function of time. The mixing time of the 1500 kg of pomace with 1000 L of water was 1 h, and the mixing temperature was 24 °C. The volume of aqueous solution produced in the decanter was 1600 L. Of this solution, 500 L were processed by nanofiltration for 14 h 30, until 375 L were permeated.

The permeate flux decreases rapidly during the first 2 h, and then more slowly, until it reaches an almost stationary behaviour, at low values. 300 L were permeated in the first 8 h, so that the yield in the last 6 h was very low. The observed permeance decrease over time is very likely due to membrane fouling (owing to adsorption at the membrane surface), although osmotic pressure effects may also be a relevant factor to consider. The fouling phenomena for nanofiltration of aqueous solutions obtained from olive oil by-products have been previously reported (Tapia-Quiros et al. 2022a, 2022b, 2022c, 2022d). One of the main factors affecting membrane fouling is the polyphenolic concentration which in this work was always maintained particularly high in order to obtain a highly concentrated product and to avoid the necessity of processing large-volume streams. However, regular cleaning procedures allowed to remove accumulated foulants and restore the trans-membrane fluxes.

For comparison, Nunes et al. (2019) measured the rejection values and the fouling behaviour of three types of membranes, two of nanofiltration and one of reverse osmosis, when contacted with a solution resulting from the water extraction of a treated olive pomace. They obtained similar results to ours with a NF270 nanofiltration membrane, although their values of fluxes cannot be directly compared with ours, because they worked with solutions obtained with water/pomace ratios of 40, and therefore much more diluted than ours.

The permeates of nanofiltration were then concentrated by reverse osmosis. This operation was much faster than the nanofiltration, yielding volume concentration factors close to 9 in approximately 2 h. In fact, the NF permeate is a highly clean feed, free from particles, colloids and macromolecules, and so it does not cause membrane fouling, being the osmotic pressure, the only factor slightly affecting the membrane flux in the reverse osmosis operation.

The results shown above prompted us to carry nanofiltration—reverse osmosis experiments using three batches of 500 L of aqueous extracts that were stopped after recovering 250 L of permeate. Reverse osmosis was performed on the 750 L accumulated with the three nanofiltrations. The final product was the reverse osmosis retentate, with volumes of approximately 80 L. In Table 1, the concentrations of the feed to nanofiltration (aqueous extract), of the nanofiltration permeate and of the reverse osmosis retentate are presented, for experiments run in 2015 (using pomace of the harvest of 2014) and 2016 (with pomace of 2015). In 2015, only the hydroxytyrosol concentration was measured, while in 2016 tyrosol was also included.

**Table 1:** Concentrations of the different process streams in HT and Ty, and ratios between concentrations of RO retentate and NF permeate and RO retentate and feed

	Feed		NF Permeate		RO Retentate		(RO retentate)/(NF permeate)		(RO retentate)/Feed	
	HT (g L^−1^)	Ty (g L^−1^)	HT (g L^−1^)	Ty (g L^−1^)	HT (g L^−1^)	Ty (g L^−1^)	HT	Ty	HT	Ty
2015	0.64	–	0.57	–	5.79	–	10	–	9	–
	0.66	–	0.55	–	4.54	–	8	–	7	–
2016	1.44	0.34	1.29	0.22	11.24	2.03	9	9	8	6
	1.68	0.43	1.48	0.30	12.69	1.94	9	7	8	4
	1.73	0.32	1.53	0.26	14.82	2.24	10	9	9	7
	2.01	0.37	1.66	0.29	15.05	2.47	9	9	8	7

Feed to Nanofiltration (Feed), Nanofiltration Permeate (NF Permeate), Reverse Osmosis Retentate (RO Retentate), Hydroxytyrosol (HT) and Tyrosol (Ty)

It should be noted, in the first place, that the final concentration in the reverse osmosis retentates depends directly on the concentration in the feed of free hydroxytyrosol and tyrosol. In fact, the derivatives of these alcohols also existent in the pomaces are retained by the nanofiltration membrane, and only the free molecules are permeated.

Table 1 shows a considerable enrichment in hydroxytyrosol and tyrosol, with the reverse osmosis retentates being on average 8 times more concentrated in hydroxytyrosol than the pomace extracts and 6 times in tyrosol. Overall, the operation could handle 1500 kg of olive pomace in a working week, yielding about 80 L of an aqueous solution containing, in the favourable case, 1.2 kg of hydroxytyrosol and 0.2 kg of tyrosol. The process uses an abundant and cheap raw-material and additional water, without need for any other solvent.

For comparison, the process developed by Sygouni et al. (2019) yields a reverse osmosis retentate with only 250 mg L^−1^ of total phenolic concentration. Bellumori et al. (2018) used also an elaborate procedure, as described above. Their reverse osmosis retentates had approximately 2 g L^−1^of hydroxytyrosol in 2013 and 2014, and 4 g L^−1^ in 2015.

Furthermore, the dry residue content was determined for our reverse osmosis retentate with the highest concentration in hydroxytyrosol (Table 1). The result was 104 g of solid per liter of liquid solution. Consequently, for this experiment, the mass of hydroxytyrosol per mass of dry residue was 15%, a promising result regarding hydroxytyrosol and tyrosol applications in pharmaceutical solid forms, since in this case, the mass of hydroxytyrosol per mass of dry residue is a crucial parameter to consider.

The highest reported value in the literature so far for reverse osmosis retentates was 10% of HT in dry basis, given by Bellumori et al. (2018).

### Stability of HT-rich concentrates

**Fig. 4:**
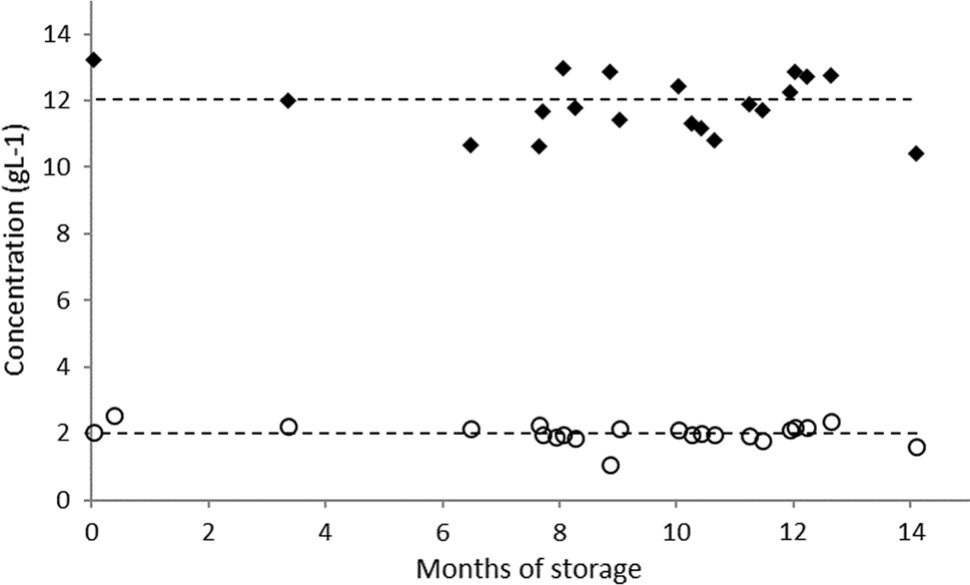
Hydroxytyrosol (HT) and tyrosol (Ty) concentration in a reverse osmosis retentate over time, at room temperature in an opaque closed container under air atmosphere

Another important factor to consider in the production of hydroxytyrosol-rich concentrates is the stability of the product (reverse osmosis retentate) over time and the eventual need for special conditions of storage. In fact, pure hydroxytyrosol used as standard in chromatographic experiments is a highly reactive material when exposed to air, and it must be used immediately after opening the air-resistant packaging where it is sold. To this purpose, reverse osmosis retentates were produced and stored at room temperature for 12 months, and the concentrations of HT and TY measured along the time. Results are presented in Fig. 4.

They show a somewhat surprising high stability of the concentration of hydroxytyrosol and tyrosol in the aqueous solution over that period of time.

Bellumori et al. (2018) reached a similar conclusion. The concentration of most of their concentrates—reverse osmosis retentates, further evaporated in some cases—remained stable over 24 months.

## Conclusion

A simple pilot-scale process was carried out for the recovery of concentrated aqueous solutions of hydroxytyrosol and tyrosol from olive oil by-products. The process consists in performing an aqueous extraction from olive pomace, followed by a nanofiltration step and a final concentration using reverse osmosis. Results obtained show that the final concentration in the reverse osmosis retentates depends directly on the concentration of free hydroxytyrosol and tyrosol in the nanofiltration feed. These concentrations varied considerably between harvest seasons and with storage time, attaining a maximum value approximately 5 months after olive oil production.

Overall, the operation could handle 1500 kg of olive pomace in a working week, yielding about 80 L of an aqueous solution containing, in the favourable case, 1.2 kg of hydroxytyrosol and 0.2 kg of tyrosol. The concentration of these solutions remained stable at least during 14 months.

## Data Availability

The datasets used and/or analysed during the current study are available from the corresponding author on reasonable request. The work described in the present paper has not been published before and is not under consideration for publication elsewhere. Its submission to JFST publication has been approved by all authors as well as the responsible authorities of the institute where the work has been carried out. If accepted, the work will not be published elsewhere in the same form, in English or in any other language, including electronically without the written consent of the copyright holder, and JFST will not be held legally responsible should there be any claims for compensation or dispute on authorship.
